# Cocaine in Vomiting of Pregnancy

**Published:** 1885-06

**Authors:** 


### Cocaine in Vomiting of Pregnancy.

Weiss, in the Centralblatt for Therapeutics, suggests the following preparation of Cocaine for vomiting in pregnancy:

| | |
|---|---|
| Cocaine Hydrochlor, . . | Grains V. |
| Alcohol, . . . . . | G'r to dissolve. |
| Water, . . . . . | Ounces 10. |

A teaspoonful every half hour.

